# Anthropogenic Influences on Macro-Level Mammal Occupancy in the Appalachian Trail Corridor

**DOI:** 10.1371/journal.pone.0042574

**Published:** 2012-08-06

**Authors:** Peter L. Erb, William J. McShea, Robert P. Guralnick

**Affiliations:** 1 Department of Ecology and Evolutionary Biology, University of Colorado at Boulder, Boulder, Colorado, United States of America; 2 Smithsonian Conservation Biology Institute, Front Royal, Virginia, United States of America; 3 University of Colorado Museum of Natural History, University of Colorado at Boulder, Boulder, Colorado, United States of America; University of Western Ontario, Canada

## Abstract

Anthropogenic effects on wildlife are typically assessed at the local level, but it is often difficult to extrapolate to larger spatial extents. Macro-level occupancy studies are one way to assess impacts of multiple disturbance factors that might vary over different geographic extents. Here we assess anthropogenic effects on occupancy and distribution for several mammal species within the Appalachian Trail (AT), a forest corridor that extends across a broad section of the eastern United States. Utilizing camera traps and a large volunteer network of citizen scientists, we were able to sample 447 sites along a 1024 km section of the AT to assess the effects of available habitat, hunting, recreation, and roads on eight mammal species. Occupancy modeling revealed the importance of available forest to all species except opossums (*Didelphis virginiana*) and coyotes (*Canis latrans*). Hunting on adjoining lands was the second strongest predictor of occupancy for three mammal species, negatively influencing black bears (*Ursus americanus*) and bobcats (*Lynx rufus)*, while positively influencing raccoons (*Procyon lotor)*. Modeling also indicated an avoidance of high trail use areas by bears and proclivity towards high use areas by red fox (*Vulpes vulpes)*. Roads had the lowest predictive power on species occupancy within the corridor and were only significant for deer. The occupancy models stress the importance of compounding direct and indirect anthropogenic influences operating at the regional level. Scientists and managers should consider these human impacts and their potential combined influence on wildlife persistence when assessing optimal habitat or considering management actions.

## Introduction

As the distribution of species shift due to recent global and local environmental changes (e.g., [1]), there is a need to determine which factors shape those distributions and at what spatial extent those factors are operating. Among the most important factors influencing animal species distributions are human activities. Such anthropogenic activities can have species-specific positive or negative effects on site occupancy and ultimately distributions. Some of these factors, such as the amount of available habitat and hunting, have a direct influence on distributions. For example, species responses to forest loss can vary depending on species-specific habitat use; forest loss often leads to decreased distribution of forest specialists [2] and increased distribution for edge habitat species [3]. Both legal and illegal harvest of wildlife can directly impact wildlife numbers and ultimately species’ distributions [4], [5]. In addition to these direct effects, humans can impact wildlife in more subtle and diffuse ways, such as mammals avoiding high-use roads [6] and trails [7]. While the impact of any one of these factors may not be detectable at the local level, when assessed region-wide, sampling may capture sufficient variation in these multiple factors, facilitating the detection of measurable and compounding effects on a species’ occurrence [8].

To study anthropogenic effects on mammal occurrence at a landscape level, we chose to focus on the Appalachian forest ecosystem [9] and, in particular, the Appalachian Trail (AT) corridor, which extends across a broad latitude of the eastern United States. The assessment of land use effects on wildlife has been identified as a significant research priority in the region [10].

Environmental variation between samples, and across landscapes, creates a major challenge to studying anthropogenic influences on species distributions [11], [12]. Utilizing the AT as a “mega-transect”, we were able to limit habitat variation between samples. The AT corridor forms a continuous habitat corridor comprised of mature, oak-dominant forest, allowing us to study anthropogenic effects while minimizing the confounding influence of heterogeneous habitat types. Because the AT passes through multiple land ownerships with diverse policies toward human activity, we are able to sample a broad range of human impacts. Although individual entities along the AT have attempted to monitor wildlife communities [13], [14], larger surveys that cross administrative boundaries have not been conducted.

Beyond the habitat-based limitations of most macro-level studies, logistical and financial challenges also often hinder researchers’ ability to achieve sufficient sample sizes. Such studies require large amounts of labor and technological equity, resources that typically limit studies to smaller extents than the science would dictate. Financial, technical and human resource constraints can be met through the use of a citizen-run camera trapping methodology. Citizen science methods have been highly successful in understanding ecological processes occurring at broad geographic extents [15]. Camera trapping is a common survey method with the capability of producing large amounts of data on the distribution and abundance of multiple mammal species [16]. Recent developments in digital camera trapping technology have improved scientific reliability, while reducing costs and simplifying setup protocols. The relatively minimal labor requirements, ease of use, and quality of data makes for an effective tool in macro-level monitoring efforts [17] and citizen science-based projects. Volunteer participation in camera trapping projects decreases costs and answers the challenge of large spatial extent, multiple species monitoring [18]. The established volunteer network used to maintain the AT corridor provided the unique opportunity to develop a citizen science wildlife monitoring project to investigate occupancy drivers through the region.

Utilizing local volunteers in this macro-level spatial study of mammal occupancy, we address the question: Do localized human activities have predictive power across the broader landscape for determining occupancy of mammals within the Appalachian Trail corridor? In answering this question, we hope to identify region-wide threats to mammal persistence.

## Methods

### Study Area

This study took place along a section of the Appalachian National Scenic Trail (AT), a protected 300 m corridor that stretches 3625 km from Maine to Georgia and is administered by the National Park Service. Though the AT corridor itself is mostly forested, it is surrounded by a mosaic of agricultural, residential, and industrial development [19]. Roughly 3–4 million people per year use the AT for recreation [20]. While hunting is not allowed in the corridor itself, adjacent lands vary in their permission of public hunting.

**Figure 1:**
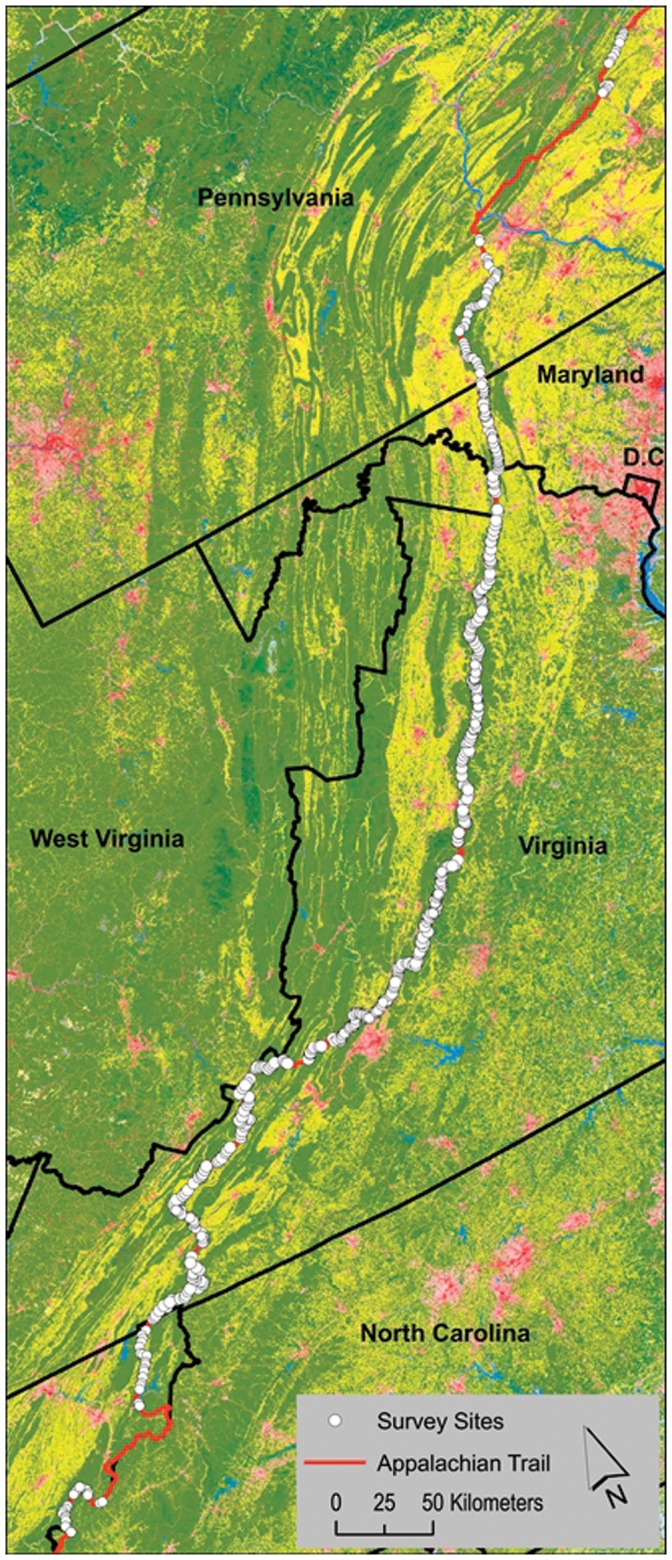
Map of study area and distribution of survey sites along the Appalachian Trail. The 2006 National Land Cover Data is used to indicate forest (green), agricultural (yellow), and urban (red/gray) land use.

We focused efforts along a 1024 km section of the AT from Pennsylvania to North Carolina (Figure 1). Data were collected over three survey years. In 2007 and 2008, the study was conducted from April to November on a 756 km segment of the AT in Maryland and Virginia. During 2009, the 2007–2008 study area was expanded to include areas to the north (Pennsylvania) and south (North Carolina, Tennessee) for an additional 268 km. This subsection represents the Appalachian-Blue Ridge forest ecosystem [9]. The forest within the study area consists primarily of Northeastern Interior, Southern and Central Appalachian Oak Forests (72%), Southern and Central Appalachian Cove Forest (18%), and other mixed hardwood forest types (<10%) [21].

### Survey Design and Sampling Protocol

The Appalachian Trail study area was divided into 2 km segments, and sample points (UTM coordinates) were randomly selected within each segment. Selected points were separated by ≥1 km and placed 50–500 meters from the trail. Land ownership of each segment was determined and permissions were obtained from the 8 public agencies involved along this segment of trail. We excluded 76 of 512 segments (15%) following consultation with NPS staff based on difficulty of access across private lands or presence of sensitive plant species.

Trail clubs responsible for each section of trail were identified and, through our partners at the Appalachian Trail Conservancy (ATC), club members were recruited to form survey teams. Two Virginia Naturalist Clubs and one local chapter of the Sierra Club also participated in volunteer recruitment. Volunteers (61 in 2007; 62 in 2008; 25 in 2009) participated in training sessions prior to each field season. They were instructed on camera placement, maintenance, file management, and sampling protocol. Teams were assigned multiple sample points and given enough cameras to complete their assignment over a 7-month (April 1– October 30) period. Cameras were left at each location for an average of 30 days. Each team was free to organize placement and movement of cameras, but remained in periodic contact with the authors and ATC staff for assistance.

Infra-red, remote-trip camera units (Cuddeback Digital; Non-Typical, Inc., Park Falls, WI, USA and Bushnell Trail Scout Digital; Bushnell Corp., Overland Park, KS, USA) were used to record presence/absence of mammal species at each sample point. Volunteer teams were given UTM coordinates and instructed to place the camera <100 m from that location and >50 m from the trail. Cameras were set on trees at knee height (<0.5 m), oriented parallel with the slope, and the viewing area was cleared of obstructions. One ml of scent lure (either MagnaGland or Pro’s Choice; Montgomery Fur, Ogden, Utah, USA) was used 1 meter in front of the camera to slow animal movement and compensate for slow camera trigger times. Digital flash cards and batteries were switched at the end of each sampling session and cameras were moved to the next survey site. Volunteers recorded location (UTM), dates, scent and lure type. When possible, cameras were checked and lure was reapplied mid-month. A National Park Service website was created for volunteers to enter data and, through the website, digital pictures on the flash cards were uploaded to an ftp server. All volunteers recorded a picture each time they setup or checked the camera to ensure its continued operation. If the camera failed to trigger, the last recorded picture was considered the last operational day [22].

### Data Compilation and Management

[LOOSESR]All uploaded photos were reviewed and subjectively graded (0–3) for quality based on orientation of camera. Sites with improper placements (restricted field of view, camera pointed too high or too low), camera malfunctions, insufficient survey time (<15 days), or incomplete data collected by volunteers were discarded. Species were identified and presence/absence data was recorded for each day in a detection history matrix following the approach of MacKenzie et al. [23].

We extracted from our GIS coverage environmental covariates that may be important for determining occupancy and detection probabilities. These covariates are listed in Table 1. Deciduous forest data was extracted from the 2006 National Land Cover Data (NLCD) for 500 m, 1 km, 3 km, 5 km, and 10 km radius buffer distances around each sample site using ArcGIS (version 9.3; Environmental Systems Research Institute (ESRI), Inc., Redlands, CA, USA). These distances were chosen after taking into account the geographic extent of the project, the variation in the home ranges between different species, and the grain of the satellite data. Hunting classifications were determined for each ownership type of adjoining land and amended to GIS ownership data provided by the National Park Service. The hunting status of land was known for public lands (81%) and was assumed to be hunted if private land. A landowner survey performed by one the authors along a segment of the AT in Virginia found 87% of private lands to be hunted. Hunting classification of adjacent land was then extracted to sample point data as hunting or non-hunting. Recreational trail use was quantified as a binary classification: high or low use. High trail use areas were identified as areas within 1 km from trailheads, in national and state parks, and further refined based on interviews of ATC members and personal observation. GIS road data was acquired from Census 2007 TIGER/Line county data sets and distances were calculated from each sample location to nearest road.

**Table 1:** Covariates used to model occupancy and detection probabilities.

Abbreviation	Name	Description
**Occupancy covariates:**		
Dec500 m	Amount Deciduous Forest (500 m)	Numeric
Dec1 km	Amount Deciduous Forest (1 km)	Numeric
Dec3 km	Amount Deciduous Forest (3 km)	Numeric
Dec5 km	Amount Deciduous Forest (5 km)	Numeric
Dec10 km	Amount Deciduous Forest (10 km)	Numeric
Road	Distance to Road	Numeric
Hunting	Hunting	Categorical (Yes, No)
TrailUse	Trail Use	Categorical (High, Med/Low)
**Detection covariates:**		
Season	Summer Season	Categorical (June–Sept.)
CamType	Camera model	Categorical (Cuddeback, Bushnell)
Lure	Lure type	Categorical (MagnaGland, Pro’s Choice)
ReAp	Reapplication of lure	Categorical (Yes, No)
Setup	Setup quality	Categorical (High, Low, Good)

Abbreviation, full name and description of data type are provided. Abbreviations serve as a reference for the species-specific model lists in Table 3.

### Data Modeling and Analysis

The modeling program PRESENCE was used to estimate occupancy (psi) and detection (p) probabilities for each species of interest (v. 2.2) [24], [25]. PRESENCE uses a logistic regression framework to estimate the probability a site is occupied by a given species. Recognizing species are not guaranteed to be detected even when present, PRESENCE uses repeated surveys and potential detection covariates to estimate the probability that a species will be detected [23]. Models are then evaluated using maximum likelihood methods.

A detection history for each site was created using the results from the repeated site surveys over the course of the three years given the aims of this study. Due to the nature of camera trapping data and the immense amount of data received, the detection history was consolidated prior to analysis to increase the statistical power of the models [22], [26], [27]. Consolidating into 5-day segments was determined as an appropriate length of time providing a balance between over-compressing and under-compressing for statistical power, while paring the dataset to a manageable size for computational purposes. Eight species were detected with sufficient frequency for convergence in PRESENCE and models for those species are considered in more detail below. For these 8 mammal species, we conducted a separate investigation of spatial autocorrelation of site occupancy using Moran’s I, where values range from 1 (a complete clustering of detections) to −1 (a negative autocorrelation). This analysis was conducted using the Moran’s I tool in the Spatial Statistics toolbox in ArcGIS (version 9.3; Environmental Systems Research Institute (ESRI), Inc., Redlands, CA, USA).

PRESENCE was then used to estimate the influence of the four sampling covariates (percent forest, presence of hunting, trail use, and distance to road) on site occupancy for each species. Multi-season modeling was used to account for colonization or extinction occurring between the sampling seasons. While utilizing this type of modeling allowed for the incorporation of colonization and extinction parameters in our models, we did not attempt to predict these parameters using covariates. The goal of this study is to determine the best predictors of site occupancy, not inter-season dynamics. Due to the strong correlation between the different scales of forest cover, each percent forest covariate was investigated independently in separate models. PRESENCE was also used to determine the effect of the 5 detection covariates on detection probabilities for each species (Table 1). To limit the number of *a priori* models examined, we used a two-step model building method based on the Cormack-Jolly-Seber data type [28]. In this approach, the first step is to hold a general occupancy model (that includes all occupancy [psi] covariates) constant and investigate all combinations of detection (p) covariates. In the second step, the resulting top model for detection probability (p) is held constant while all combinations for occupancy (psi) are investigated [29], [30], [31]. All models were ranked using Akaike Information Criterion [32] and models whose ΔAIC<2 were considered as equivalent top models. Model weights for each covariate were summed for all models to compare their relative importance for each species [33]. Covariates with summed model weights >0.5 are considered the most statistically important [34].

## Results

A sampling effort of 18 807 camera days was conducted across 447 sites. Nineteen meso and large mammal species were detected and identified, along with 5 small mammal taxa, individuals from which we were unable to confidently identify to species (Table 2). For the eight species that were detected with sufficient frequency for convergence in PRESENCE, there was no significant spatial autocorrelation (Moran’s I ranging from 0.41 to −0.03) between detections for each of the species [35]. Z-scores were found to be between −1.96 and 1.96, indicating that the data was not significantly autocorrelated within a 95% confidence level.

**Table 2:** Detection rates for 8 most common species detected in this study.

Common Name	Species	Detection Rate
White-tailed Deer	*Odocoileus virginianus*	0.826
Raccoon	*Procyon lotor*	0.405
American Black Bear	*Ursus americanus*	0.394
Virginia Opossum	*Didelphis virginiana*	0.166
Coyote	*Canis latrans*	0.120
Bobcat	*Lynx rufus*	0.098
Red fox	*Vulpes vulpes*	0.078
Gray fox	*Urocyon cinereoargenteus*	0.034

Detection rates were calculated as the proportion of camera locations at which each species was detected ((total sites occupied)/(total sites)).

Given lack of spatial autocorrelation, we determined top models for each species and summed model weights for each covariate with results listed in Tables 3 and 4, respectively. Forest cover was a significantly important feature for 6 of 8 mammal species but the scale of impact differed between species. Bobcats (*Lynx rufus*) showed a strong positive relationship and red fox (*Vulpes vulpes*), raccoons (*Procyon lotor*), and gray fox (*Urocyon cinereoargenteus*a strong negative association with the amount of deciduous forest within a 10 km radius of each site. Black bears (*Ursus americanus*) and white-tailed deer (*Odocoileus virginianus*) demonstrated a positive relationship with the amount of deciduous forest within 5 km. Forest cover was not an important predictor for opossums (*Didelphis virginiana*) or coyotes (*Canis latrans*) occupancy.

**Table 3:** Top logistic models for predicting the occupancy of eight mammal species within the Appalachian Trail corridor in 2007–2009.

Bear Model							
p(Lure, ReAp, Setup, Season, CamType)	AIC	ΔAIC	AIC wgt	No.Par.	(−2LL)	est. psi	est. p
psi(Dec5 km, Hunting, TrailUse)	2241.00	0	0.725	12	2217.00	0.4703	0.19
psi(Dec5 km, Road, Hunting, TrailUse)	2242.96	1.96	0.272	13	2216.96	0.4697	0.19
**Bobcat Model**							
**p(Lure)**	**AIC**	**ΔAIC**	**AIC wgt**	**No.Par.**	**(−2LL)**	**est. psi**	**est. p**
psi(Dec10 km, Hunting)	568.46	0	0.2295	7	568.46	0.3207	.04
psi(Dec10 km)	568.70	0.24	0.2035	6	568.70	0.3168	.04
psi(Dec10 km, Hunting, TrailUse)	569.29	0.83	0.1515	8	569.29	0.3183	.04
psi(Dec10 km, Hunting, Road)	570.41	1.95	0.0865	8	570.41	0.3273	.04
**Coyote Model**							
**p(Setup)**	**AIC**	**ΔAIC**	**AIC wgt**	**No.Par.**	**(−2LL)**	**est. psi**	**est. p**
psi(1)	–	–	–	–	–	–	–
**White-tailed Deer Model**							
**p(Season, Lure, Setup)**	**AIC**	**ΔAIC**	**AIC wgt**	**No.Par.**	**(−2LL)**	**est. psi**	**est. p**
psi(Dec5 km, Road)	4728.15	0	0.284	9	4710.15	0.8487	0.44
psi(Dec5 km, Hunting, Road)	4729.69	1.54	0.131	10	4709.69	0.8445	0.44
psi(Dec5 km, Road, TrailUse)	4730.15	2.00	0.104	10	4710.15	0.8484	0.44
**Gray Fox Model**							
**p(Lure, ReAp, CamType)**	**AIC**	**ΔAIC**	**AIC wgt**	**No.Par.**	**(−2LL)**	**est. psi**	**est. p**
psi(Dec10 km)	273.71	0	0.3409	8	273.71	0.0280	0.23
psi(Dec10 km, Hunting, TrailUse)	274.64	0.93	0.2141	10	274.64	0.0261	0.23
psi(Dec10 km, Road)	275.38	1.67	0.1479	9	275.38	0.0266	0.23
psi(Dec10 km, Hunting)	275.68	1.97	0.1273	9	275.68	0.0278	0.23
**Red Fox Model**							
**p(Setup)**	**AIC**	**ΔAIC**	**AIC wgt**	**No.Par.**	**(−2LL)**	**est. psi**	**est. p**
psi(Dec10 km, TrailUse)	432.43	0	0.3111	7	418.43	0.2443	0.05
psi(Dec10 km, Hunting, TrailUse)	433.82	1.39	0.1553	8	417.82	0.2403	0.05
psi(Dec10 km)	434.03	1.60	0.1398	6	422.02	0.2288	0.05
psi(Dec10 km, Road, TrailUse)	434.43	2.00	0.1145	8	418.43	0.2439	0.05
**Raccoon Model**							
**p(Season, Setup, Lure)**	**AIC**	**ΔAIC**	**AIC wgt**	**No.Par.**	**(−2LL)**	**est. psi**	**est. p**
psi(Dec10 km, Hunting)	2161.97	0	0.2240	9	2143.97	0.4731	0.19
psi(Dec10 km, TrailUse)	2162.68	0.71	0.1570	9	2144.68	0.4534	0.19
psi(Dec10 km, TrailUse, Hunting)	2163.12	1.15	0.1260	10	2143.12	0.4677	0.19
psi(Dec10 km)	2163.66	1.69	0.0962	8	2147.66	0.4527	0.19
psi(Dec10 km, Road, Hunting)	2163.86	1.89	0.0870	10	2143.86	0.4732	0.19

Opossum Model							
p(Setup, Lure)	**AIC**	**ΔAIC**	**AIC wgt**	**No.Par.**	**(−2LL)**	**est. psi**	**est. p**
psi(Hunting, TrailUse)	1065.06	0	0.1214	8	1049.06	0.1213	0.32
psi(Hunting, Road)	1065.96	0.90	0.0774	8	1049.96	0.1149	0.32
psi(Hunting)	1066.33	1.27	0.0643	7	1052.33	0.1153	0.32
psi(Hunting, TrailUse, Road)	1066.49	1.43	0.0594	9	1048.49	0.1195	0.32
psi(Road)	1066.63	1.57	0.0554	7	1052.63	0.1091	0.32
psi(TrailUse)	1066.90	1.84	0.0484	7	1052.90	0.1110	0.32
psi(Dec5km, Hunting, TrailUse)	1067.02	1.96	0.0456	9	1049.02	0.1207	0.32

The models are composed of both occupancy (psi) and detection (p) covariates. We list all models with a delta Akaike Information Criterion (ΔAIC)<2.00. Twice the likelihood (−2LL), number of parameters (No.par.), estimated occupancy (est. psi), and estimated detection probability (est. p) is presented for each model.

**Table 4:** The summed model weight and direction of influence for each occupancy covariate in Table 1.

Species	Model occupancy covariates			
	% Forest	Distanceto Road	Hunting	Trail use
**Black bear**	0.99 (+)*	0.27 (−)	1 (−)*	0.99(−)*
**Bobcat**	0.92 (+)*	0.29 (+)	0.58 (−)*	0.35(−)
**Coyote**	–	–	–	–
**Red fox**	1 (−)*	0.32 (−)	0.34(+)	0.64 (+)*
**Gray fox**	0.97 (−)*	0.30 (+)	0.49 (−)	0.31 (−)
**Raccoon**	0.85 (−)*	0.29 (+)	0.59(+)*	0.47 (−)
**Opossum**	0.20 (+)	0.30 (−)	0.44(+)	0.35(+)
**White-tailed Deer**	0.79 (+)*	0.71 (−)*	0.40 (−)	0.30 (−)

Summed model weights are calculated as the sum of Akaike model weights for all models including the covariate of interest.

Asterisks indicate weights >0.5.

The presence of hunting on adjoining lands was the second most common factor selected by the models. Black bear and bobcat were negatively influenced by the presence of hunting in adjoining lands. The occupancy estimate of only one meso-predator, the raccoon, was positively affected by hunting in these adjoining areas.

The third strongest predictor for mammal occupancy was the amount of recreational trail use surrounding each sample point. Occupancy for bears was negatively influenced by high trail use. Red fox was the only species positively influenced by high trail use. The influence of roads was represented in the top models for most species, but was only a heavily weighted variable for deer occupancy models. Deer occurrence demonstrated a negative correlation with distance to road.

Detection probabilities for all species were affected by all the detection covariates. The summer season (June–Sept.) had the strongest positive influence on detection for bears, raccoon, and deer. Red and gray fox both showed a negative relationship with the summer season, with higher detection probabilities during the non-summer season. Lure type showed a positive effect on all species, with Pro’s Choice lure increasing detections for all species but red fox and opossum. The reapplication of lure during the mid-month check significantly increased detection probabilities for bears, bobcats, and white-tailed deer, but decreased detection probabilities for gray fox. Camera type had an influence on all species except for coyote and red fox, with Cuddeback brand cameras having a positive association with detection. Setup quality was consistently the top variable for most species and its impact was relative to the body size. For smaller mammals (coyote, gray fox, red fox, raccoon, opossum) cameras set too high had a negative influence on detection probabilities, while for larger animals (bears, deer) lower set cameras had a negative impact. Bobcat detections were not significantly influenced by camera setup.

## Discussion

Using the AT as a mega-transect, we were able to model the relationship between species occupancy and multiple anthropogenic influences. Macro-level distribution studies have primarily focused on factors visible through readily available remote data (i.e. satellite) and have shown strong relationships between such factors and species distributions [36], [37], [38]. Our occupancy models point to more subtle anthropogenic effects occurring at varying extents, some not visible by remote sensing technology.

### Available Forest

While much of the upland Appalachian oak forest has experienced significant expansion in the past 100 years, there has been significant habitat loss at lower elevations. Nearly 83 percent of the oak forest in this region has been altered, and large patches of intact habitat are limited primarily to upland public lands [9], [10]. Since the AT generally follows the ridgelines through the region, variability in our measure of forest cover is primarily assessing loss of forest at lower elevations. Forests within the AT corridor have probably improved through public management, but our results indicate the status of forest outside the corridor impacts the distribution of our focal mammals. Numerous studies have shown the importance of the amount of available habitat to species occurrence (e.g., [39], [40]) and our results supported these findings. The amount of oak forest was the most significant predictor of occupancy for six out of the eight species.

Our results also demonstrate the impact of forest loss is species-specific. Forest loss negatively affected the occurrence of larger carnivores such as bears and bobcats, while positively affecting many meso-carnivores such as fox, and raccoon. Two species, opossum and coyote, were not drastically impacted by change in forest cover. Not only did our results show variable response to forest cover, but also in the spatial scale at which species responded to forest loss, a trend demonstrated in other studies (e.g., [41]). All our focal species responded most strongly to available forest at larger (5 or 10 km) rather than smaller scales (500 m–3 km). Such variation in scale response often depends on species traits such as body size and size of home range [42], but such possible mechanistic explanations were not investigated in this study.

### Hunting

**Figure 2:**
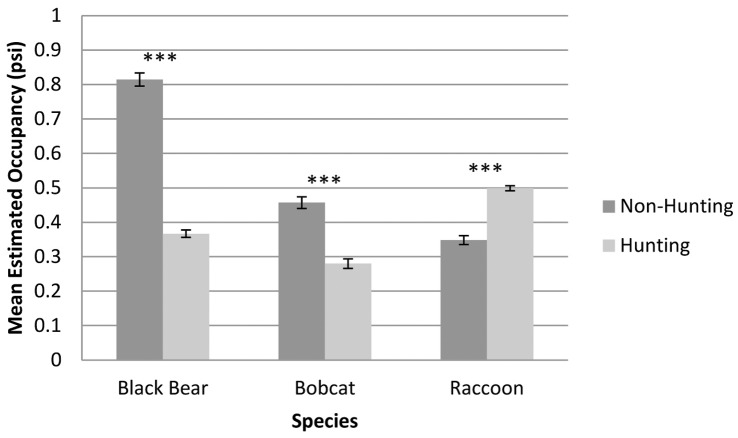
Mean and standard error of estimated occupancy in hunting vs. non-hunting areas for the 3 species for which hunting was present in the top models and received >0.5 Akaike weight. Asterisks represent level of significance based on two sample t-test assuming unequal variance (*** = p<0.001).

Hunting has a direct effect on wildlife populations and their distributions. Wildlife management of game species is based on the premise that populations can be regulated by public hunting (e.g., [43]). Our research extends this paradigm to include impacts on adjacent non-hunted areas. Hunting is a popular form of recreation through the Appalachian region and is closely regulated by state wildlife programs. The presence of hunting adjacent to the AT was the second strongest predictor for species occupancy within the corridor. While all species studied are considered game species through the majority of the AT corridor, only three out of eight were significantly influenced by hunting. One game species, bear, and one furbearer species, bobcat, were negatively impacted by hunting. Only the raccoon was positively influenced by the presence of hunting. The difference in occupancy rates in hunting versus non-hunting areas was highly significant (p<0.001) for all three species (t-tests assuming unequal variances were run for each species; Figure 2). A positive relationship between hunting and raccoon occurrence is in agreement with the meso-carnivore release hypothesis: as predation pressures decrease due to the decline in predator occurrence, smaller carnivore species, such as raccoons, may experience increases in population and an overall increase in occurrence [44], [45], [46].

### Trail Use

**Figure 3:**
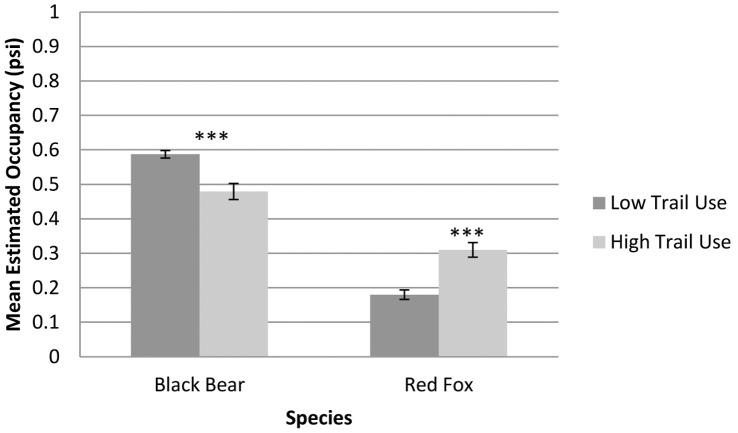
Mean and standard error of estimated occupancy in low vs. high trail use areas for the 2 species for which trail use was present in the top models and received >0.5 Akaike weight. Asterisks represent level of significance based on two sample t-test assuming unequal variance (*** = p<0.001).

Trail use is highly variable along the AT, with heavy trail use generally occurring on sections of trail that are in close proximity to large population centers, provide easy access, and/or occur in popular state and national parks [10]. With growing human populations adjacent to many stretches of the AT, understanding the effects of recreational use on wildlife is important. Work by Miller et al. [47] has shown the negative influence of trail use on a number of species. Deer and bobcats have repeatedly shown avoidance and flight behavior within short distances of heavily used trails [48], [49]. Many nocturnal species are likely to be unaffected by recreational activity, while some diurnal species such as bobcats have demonstrated temporal displacement by becoming active only after recreational activity has subsided [48]. Species that are not affected by high levels of human activity may be habituated to humans; documentation of habituated wildlife and co-existence with humans has occurred in many urban and high-use areas [49], [50]. In our study, the level of trail use was a strong predictor for two out of eight species. Our results indicated an avoidance of high-use areas by bears, and a proclivity toward high-use areas by red fox. The difference in occupancy rates in high-use versus low-use areas was highly significant (p<0.001) for both bears and red fox (t-tests assuming unequal variances were run for each species; Figure 3). Trail avoidance by black bears is consistent with the findings of Kasworm and Manley [51], however the reason for increased red fox occupancy in high-use areas is unclear. This may be an artifact of adjacent land use, rather than a direct response to trail use. High trail use areas are often adjacent to urban and residential lands, which have been found to be commonly used by red fox [52], [53]. Red fox success in human-dominated landscapes has been attributed to their omnivorous diet, which includes a proclivity towards human foods [54]. Such a response to human attractants could be responsible for increased occurrence of red foxes in high recreation use areas.

### Roads

**Figure 4:**
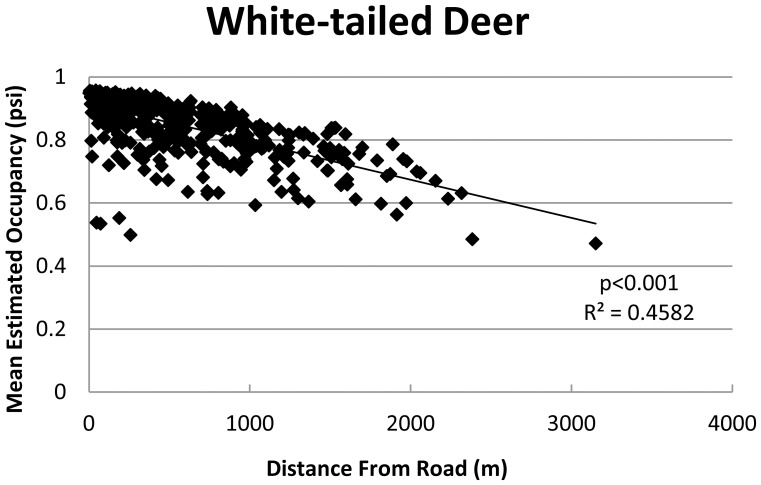
Estimated occupancy as a function of distance from road for white-tailed deer, the 2 species for which distance from road was present in the top models and received >0.5 Akaike weight.

The ecological effects of roads have been well documented. Roads can have both negative and positive effects on species occurrence and may lead to increasing habitat fragmentation, mortality, behavioral modification, and resource availability [55]. In our study, roads only predicted occupancy for deer (Figure 4). Deer attraction to roads was not surprising due to the resulting habitat modification and benefits received from increased forage [56]. Despite the effect seen in deer and the importance of roads in other studies, we did not have strong evidence that roads in our study area influenced occupancy for most species. This may be due to the dominant effects of habitat availability, hunting, and trail use, or due to highly regulated traffic speeds within the AT corridor compared to other studies.

### Management Implications and Conclusions

Our findings show that localized human activities strongly affect mammal occupancy across broad landscapes. Such findings provide an important bridge between occupancy studies at smaller spatial extents [57], and landscape level distributional modeling studies [12]. Developing further methodologies in order to incorporate both broad-scale survey and human activity data into dynamic species distribution modeling is a rapidly emerging area of research [58], [59], [60].

Our results also indicate that the compounding effects that anthropogenic factors have on wildlife cannot be ignored. While models including only roads, trail use, or hunting were never among the top model set for any species, these covariates were often represented in top multivariate models and did contribute to predictive power of overall occupancy. For example, a model representing hunting alone did not predict bobcat occupancy well (ΔAIC = 4.40), however, when considered in concert with forest cover, hunting was a meaningful predictor for this species (Akaike weight = 0.23). Minor contributions to occupancy may not appear important when assessed individually, but can exert meaningful influence when considered cumulatively [8], [61]. Land managers should recognize these important cumulative effects in management of wildlife populations.

The citizen science-based camera trapping protocol employed in this study proved highly successful for generating macro-level occupancy and local environmental data of particularly high quantity and quality. The methodology employed in this study opens up new and burgeoning possibilities for asking large scale wildlife questions that otherwise may prove impossible to answer due to logistical constraints. Our success in using this methodology not only shows the promise of such approaches for continued monitoring within the AT corridor, but for other broader extent occupancy studies around the world. Employing numerous volunteers probably affected detection probabilities for most species due to variation in camera setup quality and ability to reapply lures mid-survey, but the development of software to successfully account for these effects on detections (i.e. PRESENCE) greatly enhances the potential of both citizen science and camera trapping methodologies in macro-level studies.

A final and essential point is that the results of this study contribute to management of the Appalachian Trail corridor. The Appalachian Trail is the single most important corridor across the eastern United States. If it is to serve as a corridor between public lands for important wildlife, we must understand the attributes of an effective corridor and how to measure and monitor these attributes. While movement and connectivity were not assessed, occupancy modeling was able to identify influences on wildlife persistence within the corridor. As suggested in our results, protecting current forest habitat and encouraging continued reforestation and land acquisition would be extremely beneficial to a number of mammal species. For certain target species, hunting should be monitored closely and regulations should be adaptable in areas where occupancy might otherwise be low due to the effects of recreation and/or roads. Managers of these wildlife species should consider these factors on adjoining lands when setting management teams and actions along the AT.
